# Hospital preparedness exercises for paediatric mass casualty incidents: a systematic review

**DOI:** 10.3389/fpubh.2026.1770232

**Published:** 2026-02-26

**Authors:** Elizabeth Baxter, Zubair Ahmed, Justine J. Lee

**Affiliations:** 1 Department of Inflammation and Ageing, School of Infection, Inflammation and Immunology, College of Medicine and Health, University of Birmingham, Birmingham, United Kingdom; 2 University Hospitals Birmingham NHS Foundation Trust, Mindelsohn Way, Birmingham, United Kingdom; 3 Centre for Trauma Sciences Research, University of Birmingham, Birmingham, United Kingdom

**Keywords:** children, disaster preparedness, emergency department, mass casualty incident, paediatrics, simulation, training, triage

## Abstract

**Introduction:**

Mass casualty incidents (MCIs) present a global threat to civilians, with children often being affected and sometimes even targeted; however, there is little research regarding the preparedness exercises of healthcare professionals for such events.

**Methods:**

A systematic search of PubMed, Web of Science and Embase from inception up to July 2025, was conducted. Risk of bias was also assessed using the risk of bias in non-randomised studies of interventions exposure (ROBINS E) tool.

**Results:**

The initial search generated 223 results, and following double screening and manual citation searching, 17 observational studies were selected for narrative synthesis, since numerical data to perform meta-analysis were unavailable. The review identified a broad range of training interventions tailored for paediatric MCIs. Both brief, frequent drills and longer, mixed methods training schemes were effective, yielding gains in specific skills and a holistic sense of preparedness, including teamwork and communication. These improvements were often sustained for up to 6 months, despite a common limitation of lost to follow-up. However, the overall risk of bias in the included studies were high to very high.

**Discussion:**

MCI educational schemes appear to improve all aspects of preparedness. However, the evidence is heterogeneous, lacked standardisation in the outcome measures and contained high to very high risk of bias, suggesting that the current evidence cannot support definitive recommendations. Future research should aim to conduct high-quality studies with standardised outcome assessment tools to optimise paediatric MCI preparedness.

**Systematic review registration:**

https://www.crd.york.ac.uk/PROSPERO/view/CRD420251084048.

## 1 Introduction

Mass casualty incidents (MCIs) are defined as events that critically strain emergency service resources, necessitating the use of extraordinary measures to uphold operational standards (1). Ranging from environmental disasters to acts of terrorism, these events severely impact all facets of the emergency services, including healthcare systems (2, 3). Whilst often interchangeably used by media outlets, there is a distinct difference between MCI/and major incidents (MIs). MIs refer to any event straining emergency services. In contrast, MCIs specifically denote situations in which a high number of injuries or deaths have occurred, leading to an overwhelming impact on healthcare services due to increased patient volume (4).

Due to the significant global threat posed by MCIs, proactive preparedness strategies are necessary in every nation. For instance, the United Kingdom's sophisticated Major Trauma Network is designed to adapt to and accommodate these disasters, ensuring every patient receives the right treatment at the right time (5). In 2017 the UK experienced a series of devastating attacks, including the Westminster Bridge Attack, the London Bridge Attack, and the Manchester Arena Bombing, which pushed associated Trauma Networks to their limits. The Manchester Arena Bombing, for example, led to 160 patients needing hospital attendance, 40 of whom were children and tragically resulted in 22 fatalities (excluding the perpetrator), eight of whom were paediatric patients (6).

These incidents have spurred a wealth of literature evaluating the healthcare system's response and serving as a critical tool for identifying effective strategies and areas for improvement in MCI management (7, 8). This multifaceted literature, encompassing factual recounts and personal staff perceptions, provides comprehensive overviews that inform policymakers on potential strategies to enhance future MCI preparedness. Recurring themes in this literature indicate that despite high skill levels, staff frequently felt inadequately prepared for MCIs (7–9). In response, the NHS has since introduced a statutory framework for MCIs and MIs, integrated within the larger Emergency, Preparedness, Resilience, and Response (EPRR) guidelines (10, 11). These guidelines offer comprehensive advice on preparation and provide triage tools for both pre-hospital and hospital teams.

Preparation for MCIs involves specific tools and educational initiatives (12). Simulations are already a widely employed tool to enhance departmental working mechanisms and improve readiness for such events (13). Despite this, there is a significant gap in the literature; currently, there is no comprehensive review to synthesise the methods of paediatric MCI preparedness training. This is a critical omission as paediatric patients present unique logistical, physical, and psychological requirements that necessitate specific consideration during MCIs (14, 15). Training anecdotally treats children as “small adults” rather than addressing these unique challenges. Given that MCIs can occur anywhere, and individuals will seek care at the nearest available healthcare facility regardless of its specialisation (8), all healthcare professionals must be adequately prepared to manage such disasters.

This review aims to address this critical gap by providing a cohesive synthesis of the currently reported methods of paediatric MCI preparedness exercises. The primary objectives of this review are to identify the preparedness exercises specifically used for paediatric MCIs, their impact and their perceptions of effectiveness.

## 2 Materials and methods

### 2.1 Search strategy

This systematic review was prospectively registered with PROSPERO (https://www.crd.york.ac.uk/PROSPERO/view/CRD420251084048) and a systematic search of relevant databases was conducted following the Preferred Reporting Items for Systematic Reviews and Meta-analyses (PRISMA) statement (16). The PICO framework was used to develop the search strategy based on Table 1. A systematic search of Embase, PubMed, and Web of Science was conducted between June and July 2025. Combinations of MeSH and Boolean terms were used and following full search string was used in all databases to search for appropriate articles: ((((((((pediatric) OR (child)) OR (paediatric)) OR (neonate)) OR (infant)) OR (adolescent)) AND ((mass casualty incident) OR (mass casualty event))) AND (preparedness)) NOT (review). Following the completion of the initial search, a manual search of the reference lists of the selected sources was conducted to ensure identification of any sources not apparent on our initial search.

**Table 1:** PICO framework used to develop search strategy.

**Framework item**	**Description**
Population	Paediatric, neonate, infant, adolescent patients
Intervention	Educational simulations, drills or didactice teaching sessions
Comparator	Not applicable
Outcome	Impact of education on department or healthcare staff

### 2.2 Eligibility criteria

Studies were required to satisfy the following inclusion criteria: (1) the study population comprised healthcare staff; (2) interventions encompassed educational simulations, drills, or didactic materials (e.g., lectures, resources) that incorporated a paediatric focus or element; (3) assessed outcomes pertained to preparedness, knowledge, or the effectiveness of the drills, as evidenced by observational assessment, quizzes, or qualitative survey feedback; and (4) all included studies were published in the English language.

Studies were excluded where (1) the study population did not comprise healthcare staff. Further exclusions were applied if (2) interventions did not involve human participants or demonstrably lacked a paediatric element. Studies were also excluded if (3) their primary focus was solely on the development of an exercise or if they presented no formal assessment. Finally, (4) reports classified as case studies were excluded.

### 2.3 Study selection and data collection process

Two researchers (EB and ZA) independently screened each article using Covidence systematic review software (Covidence, Melbourne, Australia). Initial screening involved reviewing titles and abstracts. If the contents remained unclear regarding inclusion criteria, the full article was screened. There were no conflicts during the study screening process and therefore conflicts did not need to be resolved through discussion.

### 2.4 Data items

Outcomes for which data were sought included measures of preparedness, knowledge, and the effectiveness of MCI or disaster preparedness exercises. Various formats of compatible assessment were acceptable for measuring these outcomes, such as quizzes, qualitative surveys, and feedback.

A data extraction template was designed and applied to the collected data, encompassing study type, design, aim, population, and key outcomes. This ensured the identification and collection of all results aligned with the review outcomes. All included studies were compatible with all specified outcomes, although a substantial degree of heterogeneity existed in the methods by which these outcomes were measured.

### 2.5 Analytical methods

Given the expected heterogeneity of the results, a quantitative meta-analysis was not possible and a narrative synthesis was identified as the most appropriate analytical method. This approach involved the systematic organisation and categorisation of results, allowing the identification of overarching themes and patterns. Individual subgroup analyses were conducted following the categorisation of themes, allowing for deeper exploration of more nuanced ideas.

### 2.6 Quality assessment

The Risk Of Bias In Non randomised Studies of Interventions Exposure (ROBINS E) tool (17) was deemed the most appropriate method for assessing the risk of bias, primarily due to the observational nature of the included papers and their investigation of exposure effects. This is because other tools such as ROBINS-I are generally used for non-randomised studies looking at the effects of treatments, drugs or planned programs. To ensure robustness, two reviewers (EB and ZA) independently assessed the risk of bias using the ROBINS E tool; any disagreements were resolved by discussion.

## 3 Results

### 3.1 Study selection and study characteristics

**Figure 1:**
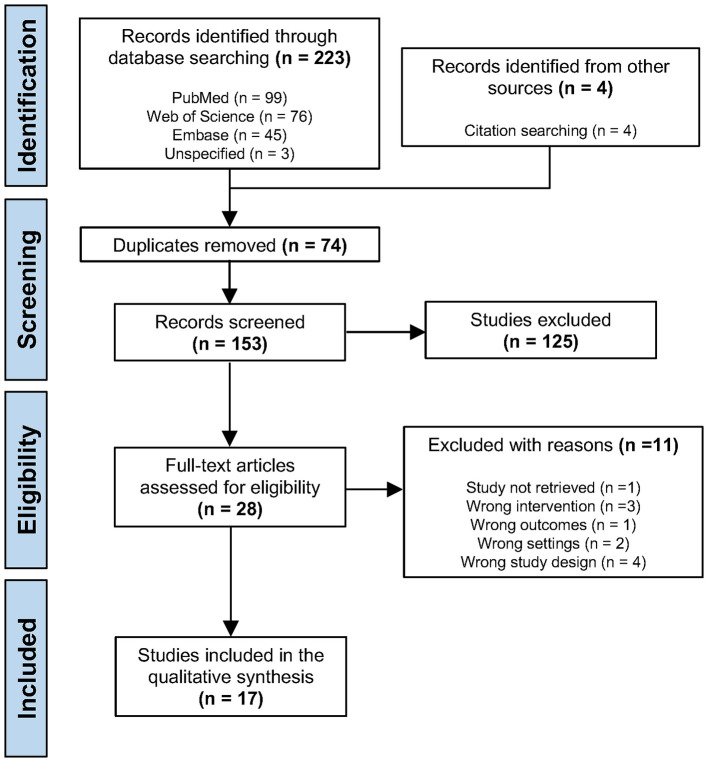
PRISMA flowchart for study selection.

An initial search generated 223 results, with an additional four studies identified through hand searching reference lists of the included studies. After removing duplicates, 153 articles were screened, from which 125 studies were excluded. Of the 28 studies that remained, 11 studies were excluded after full-text reading for various reasons including wrong intervention, wrong outcomes and wrong study design. This left a total of 17 studies that were included in this systematic review and qualitatively analysed (18–34) (Figure 1).

Fourteen of the included studies were observational (82.4%), with the vast majority [12 studies (70.6%)] conducted in the United States (20, 22–29, 31–33). Studies largely adopted single centre designs [13 studies (76.5%)], with a predominance of highly specialised paediatric trauma centres (58.8%; Table 2). Participant sample sizes also displayed a large variation, with a median of 45.5 (range 10–337) and a mean of 75.9 across the 15 studies reporting participant numbers. Mixed healthcare teams working in acute and emergency settings made up most of the samples (82.4%), with only a relatively small minority deciding to focus on specific groups of healthcare workers (19, 22, 24, 29, 34). Training programs favoured active learning, with seven studies adopting a mixed methods approach combining simulations with didactic or online teaching. The exercise scenarios were variable, ranging from hyper realistic simulations to discussion based tabletop exercises, often tailored to the type of MCI most likely to be faced in the area, such as a road traffic accident (RTA) or a natural disaster. Studies were mostly conducted between 2010 and the early 2020s, reporting a wide range of outcomes, including knowledge, skills, confidence, and teamwork. Outcomes were measured using a variety of instruments, from self-perceived Likert scales to objective assessments and external evaluations, with six studies incorporating longitudinal designs to examine knowledge retention over time (19, 23–26, 34).

**Table 2:** Summary of study characteristics.

**References**	**Study design**	**Sample size (*N*)**	**Study origin, setting**	**Exercise type**	**Outcomes measured**
Asenjo et al. (18)	Observational, case control study	28	Spain, Paediatric MTC	Simulation using high fidelity mannequins	Median time to triage, time to physician, length of stay, and proportion of patients visited
Bank and Khalil (19)	Longitudinal cohort study	27	Canada, simulation centre	Workshop including: mini plenary, clinical stations and a simulation	Subjective: retrospective pre post survey conducted after the workshop and 3 months later. Objective: evaluation of exercises.
Burke et al. (20)	Observational, cohort	Not stated	United States, 1 Level 1 paediatric trauma centre and 2 General hospitals	Full functional scale exercise	Perceived and functional readiness using, quantitative surveys, qualitative interviews and assessors during the exercises
Chou et al. (21)	Observational, cohort	49	Taiwan, paediatric hospital	Functional exercise	Perceived and functional preparedness tested by questionnaires using Likert scales
Cicero et al. (22)	Quasi experimental study	116	United States, hospital	Didactic education course	knowledge and attitudes towards PDM, experience in disaster medicine, comfort in performing PDM triage and treatment, qualitative opinion of course and attitudes to further training
Cicero et al. (23)	Longitudinal cohort study	337 enrolled, 261 completed	United States, N/A	Multi patient, multi simulation study, including pre briefing, simulation, debriefing and additional online modules	Triage accuracy and inaccuracy, learner retention, and participant characteristics
Delgado et al. (24)	Observational, Quasi experimental study	42	United States, paediatric hospital (ED)	Asynchronous online learning module and in person mass casualty incident drill	Knowledge retention, triage algorithm application, use of life saving interventions, and learner feedback
Gross et al. (26)	Observational, cohort	78	United States, paediatric hospital (ED)	Brief, *in situ* simulations, “disaster huddles”	Staff performance of critical actions and time to perform them
Hewett et al. (25)	Longitudinal cohort study	84	United States, 1 Level 1 paediatric trauma centre	Multifaceted curriculum, comprising of, didactic sessions, skills sessions, tabletop exercises, and simulations	Donning PPE, knowledge, confidence, skills retention, and participant feedback
Kenningham et al. (26)	Educational intervention and cross-sectional survey	98	United States, Conference	Didactic lectures and workshops followed by a mock MCI simulation	Mock triage scored, over and under triage rates
Li et al. (28)	Observational, prospective cohort	69	United States, 1 Level 1 paediatric trauma centre	Multidisciplinary *in situ* simulation programme (education programme followed by simulations during normal operations)	Triage accuracy, knowledge (using pre and post simulation questionnaires), self-evaluation of preparedness, and MCI skills
Marks et al. (29)	Descriptive observational study	10	United States, 1 Level 1 paediatric trauma centre	Simulation including pre and post exercise surveys and a debrief	Pharmacist performance (observer assessed), knowledge of disaster response, challenges encountered, and requests for changes/improvements (self-assessed)
Naru et al. (30)	Nonparticipant observational assessment	>200	Australia, public tertiary hospital	Large scale disaster functional exercise using the Emergo Train System (ETS)	Challenges experiences and adaptations made though nonparticipant observations and inductive analysis of field notes
Opsahl et al. (31)	Descriptive study	29	United States, multiple hospital sites	Tabletop simulation including pre and de briefing	Confidence in skills, perceived effectiveness in learning activities and impact of clinical practice, identified opportunities for improvement
Tan et al. (32)	Descriptive study	32	United States, multiple paediatric hospital (ED)	Simulation training using low fidelity mannequins, patient description cards and embedded participants	Qualitative assessment used measures such as perceived effectiveness, confidence and relevance which were assessed using Likert scales. Reflection and discussion were encouraged in the team debrief
Toida et al. (33)	Observational study	Not Stated	Japan, paediatric hospital	Triage training and a predrill education program (lecture and online education)	Efficiency of triage education, validity of START method for triage, potential need for evacuation
Wright et al. (34)	Observational, Quasi experimental study	14	United States, tertiary paediatric hospital	Quality improvement initiative incorporating the following stages: lectures, disaster-based scavenger hunt, reference card distribution and tabletop exercise	Self-reported comfort level and knowledge, attendance and engagement, balancing measures

### 3.2 Key findings and outcomes

The duration of the MCI education programs varied significantly, from very short, repetitive drills to multi-hour workshops (Table 2). Three studies employed brief interventions, with Gross et al. (26) implementing weekly “disaster huddles”, averaging at just 7 min each and led to improvement in triage accuracy over the 26 weeks. Two studies conducted single day interventions, with both showing improvements in knowledge, preparedness, and specific skills, along with positive learner feedback (21, 32). These brief interventions are highlighted as feasible and cost-effective approaches to training. Moderate duration interventions, typically 2–5 h long, also consistently demonstrated improvements. For instance, Cicero et al. (22) found that a 2 h didactic course significantly increased knowledge, though participants preferred hands on drills. Other simulation based studies of similar duration led to sustained gains in knowledge and perceived preparedness for up to 6 months. Only one study fell into the extended category with a 6 h program (34). This comprehensive approach resulted in substantial and lasting gains in self-reported comfort and knowledge, which were sustained over 6 months.

Six studies in the review used a longitudinal design to assess the durability of training effects beyond the immediate post intervention period (19, 23–26, 34) (Table 3). The follow up intervals varied between studies, with assessment periods ranging from 2 weeks to 6 months and number of data collections ranging from 2 to 26. Outcomes measured varied from objective measures such as triage accuracy (23) or the donning of PPE (25), to subjective measures of perceived preparedness (19, 34). Despite this heterogeneity, the training interventions consistently led to improvements in both perceived and objective outcomes across all studies. However, lost to follow up was a common limitation. For example, one study reported a low retest rate, with only 14 out of 42 participants completing the follow up quiz (24). Notably, some studies were able to mitigate for this potential attrition bias, as a study on HAZMAT training showed no significant difference in scores between those who completed the retest and those who did not, suggesting the findings were not skewed (25). In general, the rates for participants who completed the educational programme to the end of the course, ranged from just over 33% to just over 96% (23–26, 34), with two studies not reporting completion rates (19).

**Table 3:** Summary of longitudinal studies.

**References**	**Measurement instrument**	**Outcomes measured**	**No. of data collections**	**Data collection intervals**	**Results**	**Completion (%)**
Bank and Khalili (19)	Questionnaire using a 6-point Likert Scale	Perceived ability to manage medical and CRM components of the care of a paediatric patient in a disaster situation	2	Retrospective pre post survey format, the same questionnaire was filled out 6 months after the exercise	Confidence retained at 6 months	(not stated)
Cicero et al. (23)	Comparing participant assigned triage levels to a predetermined “Delphi gold standard” for each simulated victim	Triage accuracy	3	P0 prior to intervention, T1 2 weeks after intervention, T2 6 months after	(T2 6 months after) retainment of the 10% triage improvement. EMTs increased triage accuracy to match performance of paramedics and students paramedics declined slightly in this time	77.45
Hewett et al. (25)	32 item direct observational checklist	Number of PPE donning steps completed within 10 mins	3	Pre intervention, post intervention, 3 months post intervention	(3 month follow up) 49% increase from baseline	63.41
Wright et al. (34)	Questionnaire using a 10-point Likert Scale	To increased comfort with, and knowledge of, MCIs by PEM doctors from a baseline measurement to 8/10 on a Likert scale, and to sustain this for 6 months	3	1 month, 3 months, 6 months	Comfort 6.86/10 (6 months sustain survey), knowledge 7.0/10 (6 months sustain survey)	78.57
Gross et al. (26)	Critical actions were scored for 8 items with either, 0 (did not complete), 1 (completed partially) or 2 (completed). The total possible score for individual drills was 16	Primary: Staff performance of critical actions, the sum of the critical actions performed by the staff. Secondary: Time to performance and critical actions	26	Data collected in weekly “huddles” for a 26-week period	Cumulative effect: primary outcome (staff performance) disaster huddle scores significantly increased over time, secondary outcome (time to action) time taken to complete critical actions didn't significantly change over the study period	96.30
Delgado et al. (24)	Multiple choice quiz	EM doctors should be able to use the START and JumpSTART triage algorithms for MCIMCIs using an asynchronous model	2	Pre-test, post-test, and 4 months post simulation	4 months post test score = 73% (improvement from 49% pre-test)	33.33

### 3.3 Subgroup analysis

A subgroup analysis based on the primary goals of the exercises was conducted to identify more nuanced ideas between studies (Table 4). Three main outcome categories were identified: purely triage, a combination of triage and management and other specific elements of MCI management. The majority of the studies (*n* = 9) elected to simultaneously assess the ability of staff to triage and manage paediatric patients during an MCI (19, 20, 22, 24, 28, 31–34), in comparison to three studies focusing on triage alone (23, 27, 32). A further five focused on another specific element of MCI management, such as Hewett et al. (25) who measured the ability to don PPE (18, 25, 26, 29, 30).

**Table 4:** Summary of subgroup analysis.

**Category**	**References**	**Primary focus**
Triage	Cicero et al. (23)	To improve the accuracy of paediatric disaster triage through a multiple simulation curriculum.
	Kenningham et al. (27)	To assess paediatric MCI triage skills following a workshop and just in time training.
	Tan et al. (32)	To use a simulation based curriculum to teach the principles of the JumpSTART triage algorithm for secondary triage in an emergency department setting.
Triage and management	Bank and Kahalil (19)	To assess the effect of an experiential learning activity on the knowledge and confidence of advanced learners in a disaster response.
	Burke et al. (20)	To use mixed methods to assess the disaster response of three hospitals, with a focus on paediatric victims.
	Cicero et al. (22)	To create and implement a paediatric disaster medicine course and measure its efficacy in conveying knowledge.
	Li et al. (28)	To describe a multi modular simulation curriculum that provided exposure to triage, critical patient care, and a disaster leadership role.
	Delgado et al. (24)	To train emergency medicine doctors in the use of START and JumpSTART triage algorithms in a simulated MCIMCI.
	Wright et al. (34)	To increase mass casualty incident comfort and knowledge amongst paediatric emergency medicine doctors.
	Opsahl et al. (31)	To use a tabletop simulation with an unfolding timeline to identify opportunities to improve patient triage, interprofessional communication, and resource mobilisation.
	Toida et al. (33)	To assess the disaster preparedness of a children's hospital using a triage drill with hospitalised patients.
Other specific element(s)	Asenjo et al. (18)	To determine the impact of a disaster drill on real patients' waiting times in a paediatric emergency department.
	Gross et al. (26)	To examine if brief, “disaster huddles” could improve administrative disaster preparedness in a paediatric emergency department.
	Hewett et al. (25)	To design and evaluate a curriculum to improve paediatric ED staff skills, knowledge, and confidence in responding to a hazardous materials (HAZMAT) event.
	Marks et al. (29)	To describe a trial of pharmacist participation in a multidisciplinary paediatric emergency department disaster simulation exercise.
	Naru et al. (30)	To document challenges experienced and adaptations made during a simulated hospital disaster.

All outcomes generally focused on the acquisition of knowledge following exposure to exercises, but studies tended to approach this differently. For example, studies focusing purely on triage, such as the study performed by Cicero et al. (23) and Kenningham et al. (27) sought to demonstrate improvements in a single measurable skill, achieved through the assessment of the number of correctly triaged patients (23, 27). The same was found in studies focusing on specific elements of an MCI, such as a study by Hewett et al. (25) which evaluated skill retention in donning PPE in the case of a disaster (25). This contrasts with studies assessing triage alongside broader management skills, such as those by Bank and Khalil (19) and Burke et al. (20), who reported a more holistic sense of preparedness alongside objective measures, including the acquisition of “soft skills” like confidence in resource management and inter team communication (19, 20).

Additionally, the type of data collected varied greatly within each group. Objective, performance based tools were used across the three groups to assess outcomes. For example, Li et al. (28) and Delgado et al. (24) both measured the levels of correct triage of simulated patients (24, 25). Kenningham et al. (27) had participants self-assess their triage decisions against a correct answer key, while Cicero et al. (23) used a checklist-based tool for evaluation by an online assessor, supported by video recordings (23, 27). Another approach was employed by Toida et al. (33) who retrospectively reviewed triage tags and medical records following a drill. Knowledge based assessments were also frequently used. Delgado et al. (24) and Cicero et al. (22) both assessed triage knowledge through post-test quizzes (22, 24). Hewett et al. (25) included a multiple choice test on HAZMAT principles, and Tan et al. (32) evaluated JumpSTART triage skills using simulation based worksheets (25). Finally, self-reported scales were a popular method for gauging perceived competence. Wright et al. (34) and Opsahl et al. (31) utilised Likert scales for participants to self-assess their triage abilities (31, 34). Other studies, such as those by Tan et al. (32) and Bank and Khalil (19) incorporated follow up questionnaires for self-evaluation (19, 32).

Beyond individual performance, some studies adopted a broader approach to evaluate systemic or group level triage capabilities. Naru et al. (30), for instance, used non participant observation to analyse how teams adapted to MCI conditions and managed patient triage (30). Burke et al. (20) employed a mixed methods design, combining qualitative, and quantitative feedback with observer findings to assess systemic issues (20). Other studies indirectly assessed triage, such as Asenjo et al. (18), which measured the impact of a drill on real patients' waiting times in a paediatric emergency department (18).

### 3.4 Risk of bias assessment

**Figure 2:**
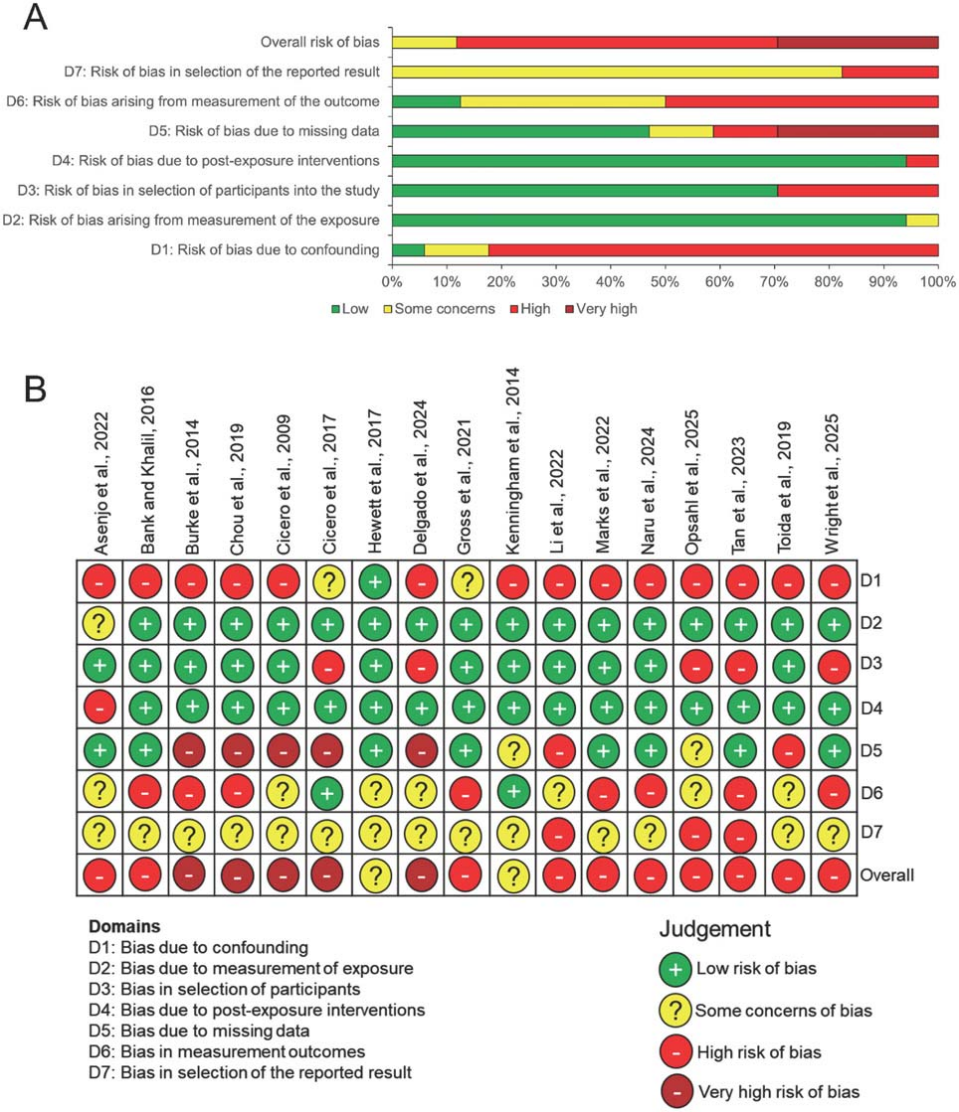
Risk of bias analysis using the ROBINS E tool. **(A)** Summary chart showing the proportion of studies with risk of bias against the seven domains and **(B)** Risk of bias in individual studies against different domains using the “traffic-light” system.

A summary chart and the risk of bias (RoB) in the individual studies are presented in Figures 2A, B. Just over 88% of the studies had an overall “High” to “Very high” RoB, with the remaining 12% of studies also demonstrating some concerns (Figure 2A). The most prevalent source of “High” RoB was Domain 1 (Bias due to confounding), affecting 14 out of the 17 included studies (Figure 2B). This was primarily due to a lack of adjustment for key confounders such as variations in the training and prior experience of staff, reliance on self-reported outcomes and the absence of control groups in the studies. The highest rate of “Very high” RoB was found in Domain 5 (Bias due to missing data), impacting almost 30% of the studies (Figure 2B). Often, this was caused by the failure of the longitudinal studies included to perform complete case analysis or adequately adjust for missing data in follow up cohorts (20, 24). One notable example was a multi hospital study undertaken by Burke et al. (20), where a disproportionately high follow up response rate from a paediatric centre led to significant data gaps from other centres, introducing a substantial risk of bias (20).

## 4 Discussion

This narrative systematic review is the first to synthesise existing literature on paediatric MCI preparedness education and exercises in healthcare. The primary objective was to identify and assess current training strategies, their impact on staff knowledge and skills, and how they were perceived by participants. The limited and varied nature of the literature available resulted in a relatively low quality of evidence. Despite this, our review identified key observations and concluded that educational preparedness schemes are an effective method of preparing healthcare workers for paediatric MCIs. Studies unanimously reported participants displaying an increased MCI preparedness through both subjective and objective measures. The existing research suggests that active learning is preferred over passive approaches (22), but it is not clear as to the relative cost effectiveness of mixed vs. active learning approaches.

This systematic review has highlighted significant variation in the duration of the educational exercises. This suggests that the length of time required for MCI training to be impactful is variable and therefore not constricted to a singular time frame. For example, for busy hospital departments looking to improve specific elements of MCI response, such as initial preparations following the notification of an MCI, a “little and often” approach may be the most suitable, such as the weekly “disaster huddles” carried out by Gross et al. (26). Alternatively, for departments where there is a large variability in staff knowledge, a slightly longer and more labour intensive mixed methods curriculum involving didactic teaching and simulations would likely be more suitable.

Most studies were conducted in hospital departments and included multiple members of the multidisciplinary team (MDT). Qualitative feedback from these exercises indicated that this approach created a realistic environment, improving skills such as communication (19, 20, 28, 30, 31). Strong teamwork and communication have been highlighted as an essential component of effective MCI response, where collaboration between departments has aided efficient and effective outcomes for paediatric patients (7). Most studies chose to focus primarily on the response of emergency departments, as this is likely to be the fulcrum of an MCI response. However, since MCIs, by their nature, overstretch a healthcare system's resources, they often require reliance on other wards and nearby hospitals (7). Consequently, studies involving the whole hospital (30), or those considering the transfer of patients to nearby facilities (33), are likely to be more useful to healthcare teams.

Recounts of management of paediatric MCIs have emphasised the unique challenges that children present to MCIs (7, 8). Many of the studies were highly targeted towards children, featuring tailored scenarios and highly specialist manikins or simulated child patients. Furthermore, specific areas of paediatric MCI response, such as the use of the JumpSTART triage tool, were explicitly tested in 10 studies (20–24, 27, 28, 31–33); the JumpSTART triage tool has now been superseded by newer triage tools such as the UK NHS MITT (35). However, the adaptations and considerations required for children extend beyond a triage algorithm. Following the Manchester Arena Bombing, several novel strategies, such as the concurrent treatment of children and parents, were recognised as effective measures for managing paediatric MCIs (7).

Although several of the included studies took into account additional adaptations, like the implementation of a hospital specific paediatric incident command structure (34), the specific nuances of paediatric patients did seem to overwhelm some centres. For instance, one study noted children were left unattended in waiting rooms, treatment and triage centres (20). Logistical challenges such as these are unique to children and were seldom considered in the studies. Jenner and Piscitelli (8) emphasised the importance of all hospitals being able to treat children, regardless of whether they are a paediatric centre (8). The low response rate from general trauma centres in a multi hospital drill, in comparison to the paediatric major trauma centre (MTC), indicates that the importance of all hospitals being able to provide a centre for paediatric patients is not being universally taken seriously (20). Future research should therefore aim to think beyond triage, encompassing all of the additional challenges that children may present with during an MCI.

Ultimately, the heterogeneity of the literature, marked by a wide variety of study designs and outcome measures, including subjective self-reported scales and objective performance based checklists, makes direct comparisons challenging. Whilst many studies successfully demonstrate an increase in knowledge and confidence following the implementation of interventions, the lack of standardisation across this field of interventions means that a conclusive analysis cannot be drawn.

### 4.1 Strengths and limitations

Despite the low quality of the available evidence, this review does have several notable strengths. A comprehensive search strategy was employed to include a broad and diverse range of study designs and outcomes. This allowed for a detailed characterisation of the study characteristics, interventions, and outcomes. Furthermore, we conducted a subgroup analysis to highlight and explore the specific nuances of groups, which helped to uncover patterns that might otherwise have been missed.

However, as discussed, the overall quality of evidence in this field is poor, with significant bias present throughout. The large and heterogeneous study designs and outcomes prevented us from completing a meta-analysis. Additionally, the results relied heavily on self-reported outcome measures, which introduced a large amount of reporting bias. Several studies even noted the drawbacks of using scales like Likert scales due to this associated bias. A disproportionate number of the included studies were conducted in the United States, which introduces the possibility of geographical bias due to differences in healthcare systems. Geographical bias is recognised in academic publishing, which is dominated by Western Europe, North America, and the Global North. This can lead to disparity in representation and access to research, particularly from low- and middle-income countries or the Global South. However, strategies to diversify academic publishing and commitment by Journals and publishers will help tackle these inequalities (36, 37).

Restricting the retrieval of studies to only those reported in English introduces language bias, geographical bias, incomplete evidence base, reduced generalisability and potential over- and under-estimation of effects, limiting the usefulness of our study. Generalisability was further limited because most of the studies were conducted at a single site. Statistically, with only one study conducting a power calculation (25), the small sample sizes of many studies likely led to underpowered results. Finally, many of the studies only went as far as assessing triage accuracy. While an important component, several other techniques are required to handle MCIs with children. Therefore, drills incorporating whole hospital systems and their surrounding areas would likely improve preparedness, as other necessary measures, such as forming a “creche” for non-injured or very minorly injured children if they become separated from parents and safely discharging patients from wards to free up beds, could also be practised.

A mixture of quantitative and qualitative outcome assessment measures was used by the studies in this review. All outcomes assessed indicated positive improvements in outcomes compared to their baseline. However, due to a large amount of heterogeneity between results, a meta-analysis was not possible; therefore, standardisation of outcome measures is required to compare methods and assess outcomes. Knowledge based assessments, such as multiple choice pre- and post-test quizzes, are a simple and relatively inexpensive method of objective assessment utilised by several studies (22, 24, 25, 28). These have no risk of reporting bias, which methods such as Likert Scales are liable to (25, 32). However, as with any self-motivated assessment method, they are liable to attrition bias, especially when measuring long term knowledge retention (22, 24, 25). Qualitative data also forms an essential component of the data collection process due to its unique ability to provide deeper insight into the perceptions of the exercises. For example, the studies conducted by Burke et al. (20) and Li et al. (28) both revealed unique insights about “soft skills” such as communication during the exercises, something that was not captured in quantitative feedback (20, 28). Future studies adopting a standardised approach that incorporates qualitative and quantitative feedback are likely to provide more robust information to this field.

### 4.2 Implication of the results for practice, policy and future research

This review has highlighted several techniques that, if implemented, have the potential to improve future paediatric MCI preparedness. The findings indicate that active learning techniques, such as drills and simulations, are associated with increased participant confidence and a heightened sense of preparedness for paediatric MCI situations, which contrasts with passive learning approaches. The review also suggests that a “one size fits all” approach to preparedness drills may not be optimal. While full scale exercises using high fidelity mannequins show positive results, smaller, more frequent exercises, such as the “disaster huddles” described by Gross et al. (26), also appear promising (26). Furthermore, the involvement of multiple members of the MDT may also more accurately mimic the teamwork required during an MCI, leading to improved preparedness.

Several papers identified their approach to MCI preparedness training as being “cost effective” (8, 21, 24, 26) for various reasons linked to their exercise format. For example, Gross et al. (26) deemed the use of “disaster huddles” a “low effort, low time commitment” resolution to a more resource intensive full scale exercise (FSE). Chou et al. (21) also made a similar argument for functional exercises (21, 26). Delgado et al. (24) highlighted the flexibility and therefore cost effectiveness that comes with combining an asynchronous online learning module with an in person simulation (35). Li et al. (28) specifically designed a simulation that required few resources and used high fidelity mannequins instead of actors to help reduce costs associated with the exercise (28). Cost effectiveness is an important consideration for any type of training within healthcare systems and maximisation of benefit to users is essential. Whilst studies may deem themselves to be “cost effective”, the lack of direct comparison with other preparedness exercise types somewhat discredits the arguments of authors. Therefore, it is imperative to conduct further research, such as randomised control trials (RCTs) to definitively assess the cost effectiveness of the preparedness exercises.

To advance the evidence base, there is a clear need for more rigorous studies, particularly RCTs. Future research should aim to compare the outcomes of purely active learning interventions with mixed modality approaches to definitively assess differences in perceived preparedness and other relevant outcomes. Such studies should use standardised outcome assessment tools to enable comparisons of how effective different interventions are. Key areas to investigate include the cost effectiveness of various education types and their impact on long term skill retention and knowledge acquisition. To inform resource allocation and optimise training effectiveness, a cost benefit analysis comparing these different modalities would be highly beneficial, considering factors beyond immediate preparedness, such as logistical feasibility. While variation in exercises may be preferable, the development of a standardised MCI educational curriculum framework, outlining feasible training timelines and structures and defining measurable outcomes, could facilitate the formation of more efficient and comparable training initiatives. Implementing an iterative feedback loop, where insights from training evaluations and drills directly inform curriculum development, would foster continuous improvement and positive change in preparedness education. Such research will provide the causal evidence needed to optimise paediatric MCI preparedness training and ultimately improve outcomes for children in mass casualty events.

## 5 Conclusion

This review underscores a critical need for the development of higher quality evidence and standardised national guidelines in the UK to enhance hospital preparedness for paediatric MCIs. Children present unique physiological and psychological challenges in MCI scenarios, demonstrated through characteristics such as smaller size and a potential inability to effectively communicate their symptoms. Therefore, regular specialised training and a tailored approach to emergency response are justified to help familiarise staff with these nuances. This review has identified several successful training techniques, ranging from table top to full scale exercises, which have been shown to improve preparedness. By implementing comprehensive guidelines and fostering rigorous, evidence-based training, the UK can significantly improve its capacity to effectively manage paediatric MCIs.

## Data Availability

The original contributions presented in the study are included in the article/Supplementary material, further inquiries can be directed to the corresponding author.
